# Biomechanical effects of a novel anatomic fusion cage for anterior cervical corpectomy: a finite element study

**DOI:** 10.1186/s12891-026-09814-w

**Published:** 2026-04-11

**Authors:** Zhanglin Wu, Yuzhu Wang, Zhijun Li, Zhiwei Lin, Hanbin Ouyang, Bo Wei, Biru Liang, Yufeng He, Wenhua Huang

**Affiliations:** 1 Orthopaedic Center, Affiliated Hospital of Guangdong Medical University, Zhanjiang, China; 2 Department of Orthopaedics, The Fifth Affiliated Hospital of Southern Medical University, Guangzhou, China; 3 Department of Orthopaedic Surgery, Zhongshan Hospital of Traditional Chinese Medicine Affiliated to Guangzhou University of Traditional Chinese Medicine, Zhongshan, Guangdong China; 4 Department of Orthopaedics, The General Hospital of Western Theater Command, Chengdu, China; 5 Department of Traditional Treatment Center, Zhongshan Hospital of Traditional Chinese Medicine Affiliated to Guangzhou University of Traditional Chinese Medicine, 3, Kangxin Road, Xiqu District, Guangdong Zhongshan, China; 6 Guangdong Engineering Research Center for Translation of Medical 3D Printing Application, Guangdong Provincial Key Laboratory of Digital Medicine and Biomechanics, National Key Discipline of Human Anatomy, School of Basic Medical Sciences, Southern Medical University, Guangzhou, China; 7 Xinyuan South Community, 2, Wenming East Road, Xinyuan Sub-district, Xiashan District, Zhanjiang, Guangdong 524005 China

**Keywords:** Anterior cervical corpectomy, Finite element analysis, Novel anatomic fusion cage

## Abstract

**Background:**

Anterior cervical corpectomy and fusion (ACCF) is a crucial surgical procedure for addressing cervical degenerative diseases. However, traditional titanium mesh cages (TTMC) are susceptible to complications, such as cage subsidence and internal fixation failure. In this study, we developed a novel anatomic titanium mesh cage (NATMC) and assessed its biomechanical properties through finite element analysis.

**Methods:**

A conventional three-dimensional finite element model comprising cervical vertebrae C2–C7 was established using computed tomography (CT) data. Subsequently, two surgical models (TTMC and NATMC) were generated based on the normal model. Uniform load conditions were applied to these distinct surgical models to simulate flexion, extension, lateral bending, and axial rotation of the cervical spine. The range of motion (ROM), stress peaks at the titanium mesh-endplate interface, screw–bone interface, and the adjacent intervertebral disc were recorded and compared between the two groups.

**Results:**

In comparison to the intact model, the surgical segmental range of motion (ROM) for C4-6 was reduced by 96.89% in NATMC and 96.91% in TTMC. The stress peaks at the C4 inferior endplate and C6 superior endplate were higher in TTMC, measuring between 1.61 and 9.96 MPa and 1.46–14.29 MPa, respectively, followed by NATMC with values of 0.93–7.75 MPa and 1.08–11.69 MPa. Stress peaks on NATMC were lower (68.47–309.90 MPa) compared to TTMC (86.80–368.30 MPa). Regarding stress peaks at the screw-bone interface, NATMC exhibited lower values (11.91–42.49 MPa) compared to TTMC (35.49–153.00 MPa). Finally, comparing stress peaks in adjacent discs (C3/4 and C6/7), NATMC and TTMC showed similar results, ranging between 1.56 and 5.47 MPa in C3/4 and 1.56–2.91 MPa in C6/7, respectively.

**Conclusions:**

The novel anatomic titanium mesh cage demonstrated effective reduction in vertebral subsidence and minimized pressure on the internal fixation interface following anterior cervical corpectomy and fusion (ACCF). It demonstrated superior stability and safety compared to traditional titanium mesh cage.

## Introduction

Anterior cervical corpectomy and fusion (ACCF) is a well-established surgical intervention for managing cervical pathologies, including ossification of the posterior longitudinal ligament (OPLL), cervical spondylotic myelopathy (CSM), and anterior spinal cord compression syndromes [1–4]. By enabling direct decompression and structural stabilization, ACCF achieves high fusion rates and remains a mainstay in complex cervical spine reconstruction [5, 6]. A critical determinant of postoperative success lies in vertebral body reconstruction, which must balance biomechanical stability with biological integration to avoid complications such as graft subsidence or hardware failure [7–9]. While autologous and allogeneic bone grafts were historically preferred, their clinical utility is constrained by donor-site morbidity, limited availability, and immunological concerns [10]. Consequently, titanium mesh cages (TMCs) have emerged as a dominant reconstruction modality due to their biocompatibility, mechanical robustness, and capacity for osseointegration [11–14].

Traditional titanium mesh cages (TTMCs), when augmented with anterior plating and bone grafting, provide immediate stability and favorable fusion outcomes [12, 13]. However, clinical studies increasingly report complications linked to TTMC use, including cage subsidence (15–40%), plate fractures, and screw loosening, which compromise long-term surgical success [15–18]. These complications are attributed to inherent biomechanical inefficiencies in TTMC design. Specifically, the limited contact area between TTMCs and vertebral endplates creates focal stress concentrations, predisposing to subsidence while amplifying mechanical loads on adjacent fixation hardware [19]. Furthermore, the non-anatomical geometry of TTMCs fails to replicate native vertebral morphology, exacerbating stress-shielding effects and impeding load distribution across the reconstructed segment.

To address these limitations, we propose a novel anatomic titanium mesh cage (NATMC) engineered to optimize endplate contact geometry and stress distribution. Leveraging patient-specific anatomical data, the NATMC design aims to mitigate subsidence risk by maximizing interface congruity while reducing stress concentrations at the cage-endplate and screw-bone interfaces. This study employed finite element analysis (FEA) to systematically evaluate the biomechanical performance of NATMC versus TTMC in a validated C2–C7 cervical spine model. Key parameters, including range of motion (ROM), stress distribution at critical interfaces, and adjacent segment mechanics, were analyzed under physiological loading conditions.

## Materials and methods

### CAD model of the intact cervical spine

**Fig. 1:**
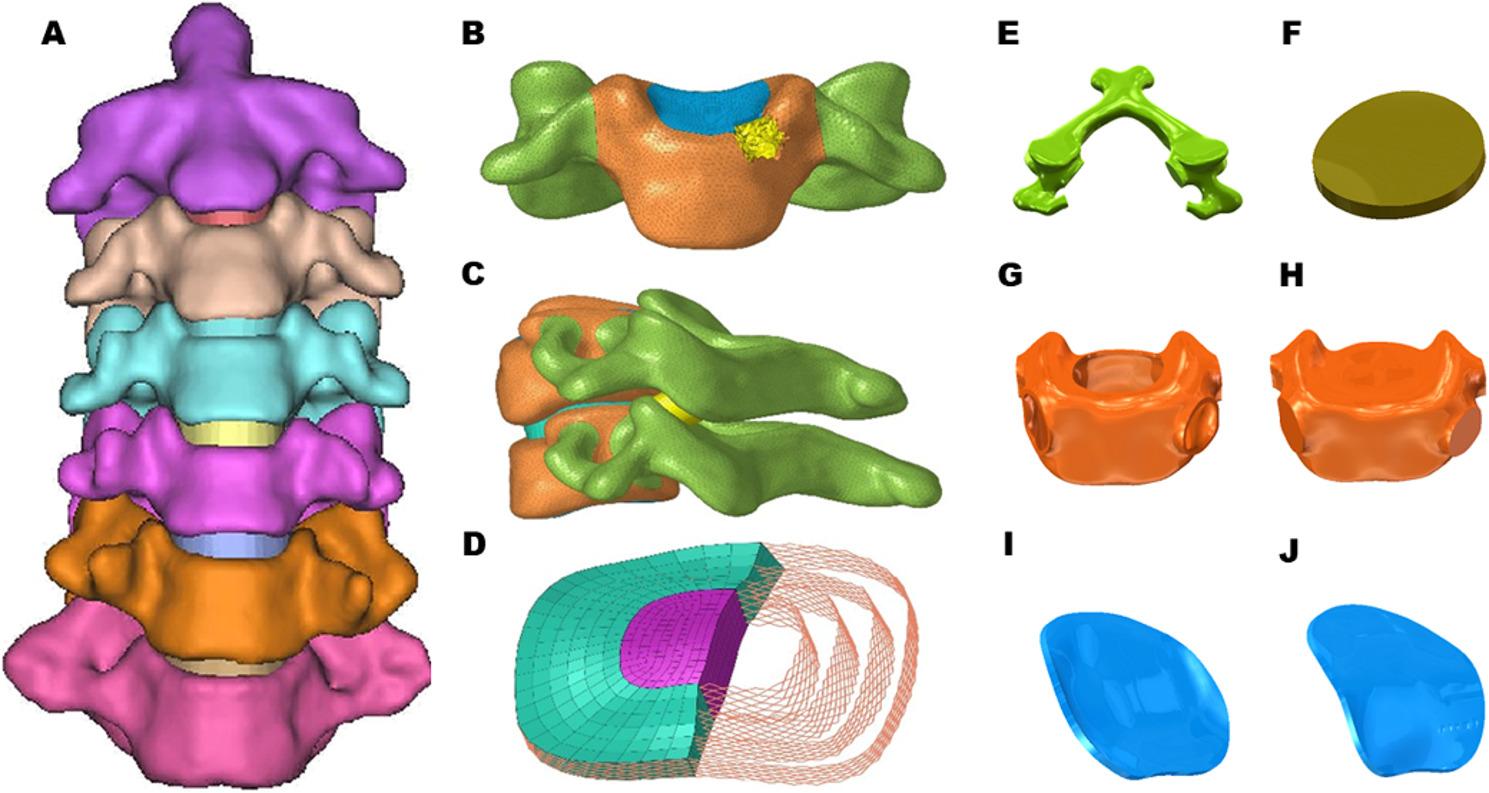
CAD model of the C2-C7 intact cervical spine, (**A**) Front view of the intact cervical model of C2-C7, (**B**) Complete vertebral structure, (**C**) Side view of the facet joint, (**D**) Intact intervertebral disk, (**E**) Posterior bone, (**F**) Cartilage, (**G**) Cortical bone, (**H**), Cancellous bone, (**I**) Upper endplate, (**J**) Lower endplate

The study was approved by the ethics committee of the Fifth Affiliated Hospital of Southern Medical University (NO. 2023BL021), the clinical trial number: not applicable. The cervical spine FE model data for this study were derived from a middle-aged female volunteer (32 years old, 52 kg, 160 cm) without cervical disease, and the volunteer provided signed consent before the study was conducted. Cervical computed tomography (CT) images were obtained in the horizontal position with a scan interval of 0.625 mm. The CT data in DICOM format were imported into MIMICS 14.0 (Materialise Corporation, Belgium) for 3D reconstruction and segmentation of vertebrae to obtain six vertebral body models of C2-7. Subsequently, the vertebrae models were imported into reverse engineering software (Geomagic Studio 2021; Geomagic, NC, USA) for model optimization and conversion to solid models (Fig. 1A).

The Cancellous bone, cortical bone, post bone, endplate, intervertebral disc, and facet joint models were generated based on solid models in SolidWorks (2021, SOLIDWORKS Co, USA). The anterior and middle columns of the vertebrae are divided into two parts: cortical bone and cancellous bone, while the posterior column is defined as post bone. The cortical bone was constructed with a thickness of 0.5 mm, and the endplate was separated from its upper and lower ends (Fig. 1B, C). The intervertebral disc was divided into two parts: annulus fibrosus and nucleus pulposus, with the nucleus pulposus accounting for about 30% of the total volume of the disc. The annulus fibrosus was modeled as an annulus ground substance embedded with annulus fibers. These fibers surrounded the ground substance and inclined to the transverse plane between 15°and 45° [20, 21] (Fig. 1D). Moreover, the structures of cartilage, posterior bone, cortical, cancellous, endplate, were modelled (Fig. 1E, F, G, H, I, J).

### CAD model of the implanted devices

**Fig. 2:**
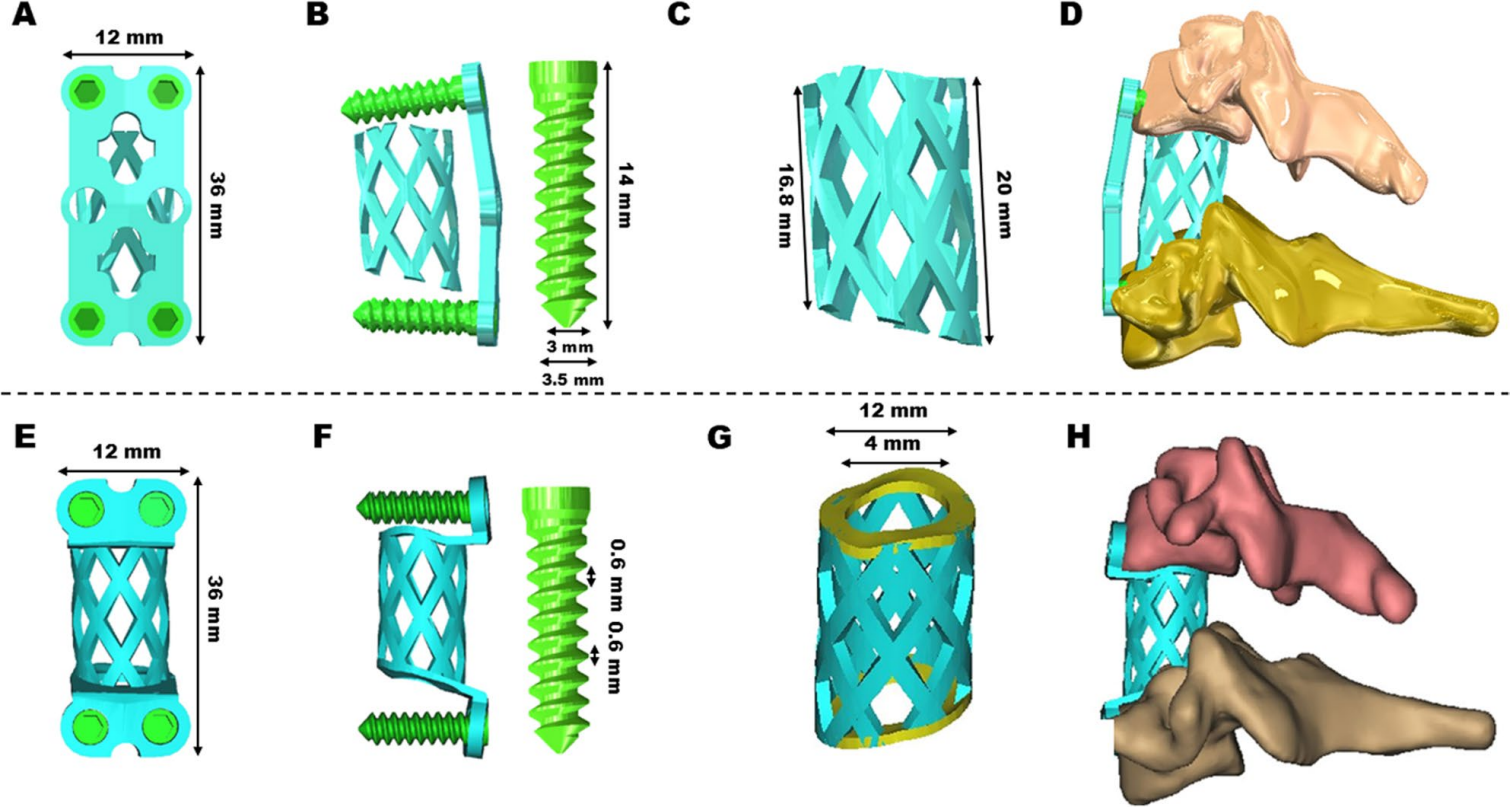
CAD models of the titanium mesh cage, (**A**) Front view of the TTMC, (**B**) Side view of the TTMC, (**C**) The mesh structure of the TTMC, (**D**) Side view of ACCF using the TTMC. **E** Front view of the NATMC, (**F**) Side view of the NATMC, (**G**) The anti-subsidence ring structure of the NATMC, (**H**) Side view of ACCF using the NATMC

TTMC utilizes a conventional titanium mesh with a 12 mm diameter. The length of TTMC is determined based on the measured distance between the lower surface of the C4 inferior endplate and the upper surface of the C6 superior endplate. The ends of TTMC are curved to align consistently with the corresponding endplate surface. The anterior titanium plate has dimensions of 36 mm in length and 12 mm in width, and the screws have a length and diameter of 14 mm and 3.5 mm, respectively (Fig. 2A, B, C, D).

The NATMC comprises a centrally positioned anti-subsidence titanium mesh and fixed ear-shaped plates at both ends. The anti-subsidence titanium mesh features a hollow column structure designed in accordance with human biomechanics, and its end part incorporates an anti-subsidence ring design. The surfaces of the anti-subsidence ring are meticulously matched with the adjacent bone endplate surfaces. The upper and lower ear-shaped plates are tailored to the surfaces of the corresponding vertebral bodies, each with a length of 36 mm and a width of 12 mm. Additionally, the screw lengths and diameters are 14 mm and 3.5 mm, respectively (Fig. 2E, F, G, H).

### Surgery simulation of ACCF

To accurately simulate ACCF surgical procedures, a corpectomy of the C5 vertebral body was performed. The excised region encompassed the vertebral body within the bilateral uncinate joint, the adjacent intervertebral disc, endplate, as well as the anterior and posterior longitudinal ligaments [22, 23].

**Fig. 3:**
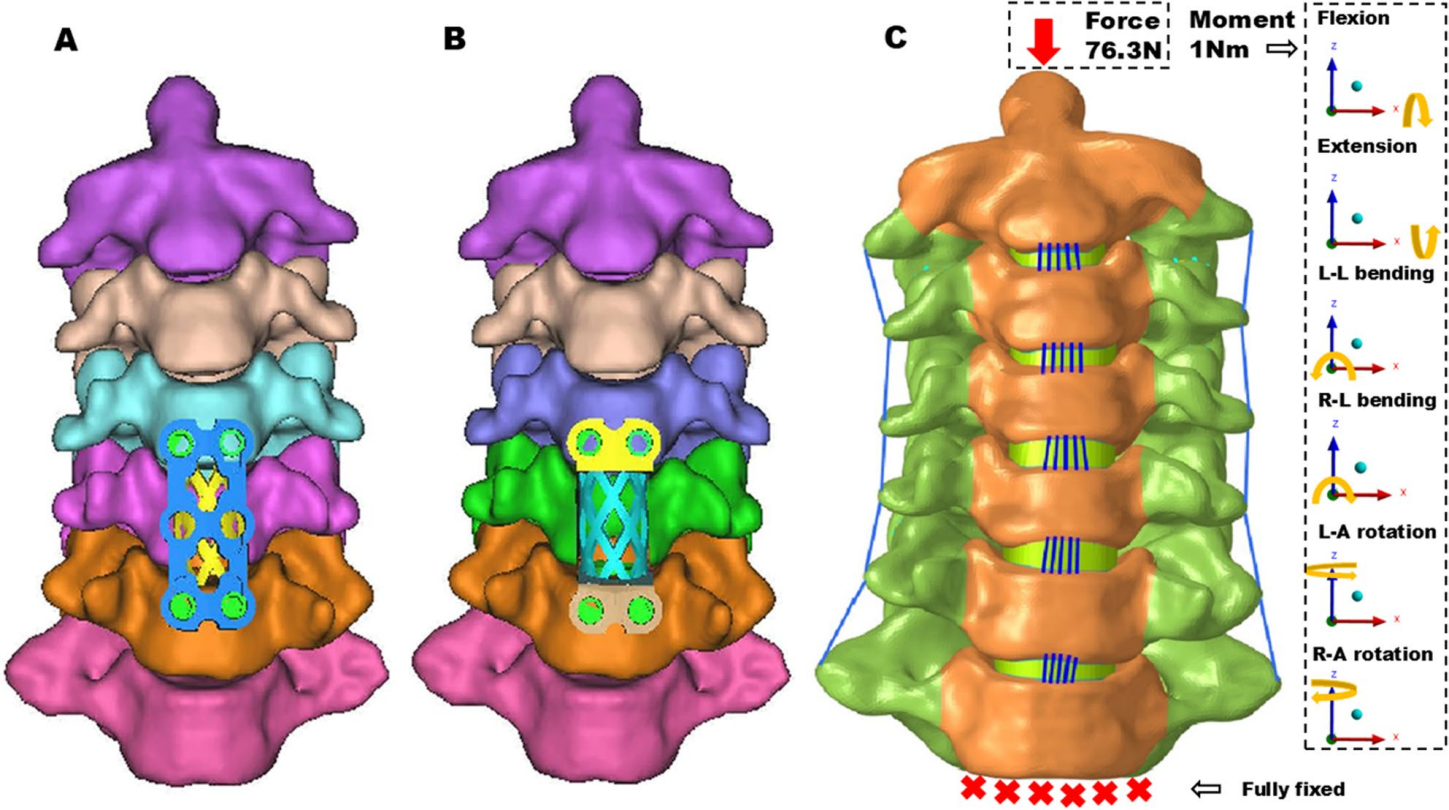
Three-dimension surgical and finite element models of ACCF using TTMC and NATMC, (**A**) ACCF using the TTMC, (**B**) ACCF using the NATMC, (**C**) Finite element model used in the study

The TMC device was positioned between the C4 inferior endplate and the C6 superior endplate, with four screws fixed inside the C4 and C6 vertebral bodies along the direction of the screw holes in the device model. The TTMC group utilized the traditional cage with a plate (Fig. 3A), while the NATMC group employed the personalized titanium mesh cage (Fig. 3B).

### Surgery simulation of ACCF

The document was then imported into FE preprocessing software (Altair HyperMesh 2021; Altair, MI, USA) to construct the FE intact cervical spine model of C2–C7. To validate the intact FE model, a pure moment of 1 Nm was applied to C2 to simulate spinal motion. The inferior surface of C7 was fully constrained with six degrees of freedom. Subsequently, the segmental ROM in flexion-extension, lateral bending, and axial rotation was compared with data obtained from previously published studies [24].

The validated model underwent analysis using FE software (Abaqus 2021, SIMULIA Co, USA). The facet joint is modeled with upper and lower articular surfaces, and the articular contact relationship with the synovial joint is defined as face-to-face, nonlinear, frictionless sliding contact [25]. The peri-vertebral ligaments, including the anterior longitudinal ligament, posterior longitudinal ligament, intertransverse ligament, ligamentum flavum, synovial ligament, interspinous ligament, and supraspinous ligament, were built with three-dimensional linear contact link elements, applying only to the tension force, as per previous study [26]. The element types and material properties used in the FE model (Table 1), based on previous publications [27–29].

**Table 1:** Material properties assigned to the finite element model

Components	Element type	Young’s modulus (MPa)	Pisson’s ratio	Cross section area (mm^2^)
Cortical	C3D4	12,000	0.29	
Cancellous	C3D4	450	0.29	
Post bone	C3D4	3500	0.29	
Cartilage	C3D8	10.4	0.4	
End plate	C3D8	600	0.4	
Nucleus pulposus	C3D8	1	0.49	
Annulus ground substance	C3D8	3.4	0.4	
Annulus fibrosus	T3D2	450	0.3	
Anterior longitudinal	T3D2	30	0.3	6.1
Posterior longitudinal	T3D2	20	0.3	5.4
Capsular	T3D2	20	0.3	46.6
Intertransverse	T3D2	20	0.3	5
Flaval	T3D2	1.5	0.3	50.1
Interspinous	T3D2	1.5	0.3	13.1
Supraspinous	T3D2	1.5	0.3	5
Titanium alloy	C3D4	110,000	0.3	

In the finite element analysis, the six degrees of freedom of the C7 inferior endplate were completely constrained. A pure moment of 1 Nm, along with a follower load of 73.6 N to simulate head weight and muscle force, was applied to C2 for the intact and ACCF FE models [30] (Fig. 3C). The ROM of the surgical segment, as well as the distribution of von Mises stress on the screw-bone interface, adjacent intervertebral disc, and TMC-endplate interface, were recorded and analyzed. These measures were utilized to evaluate the biomechanical properties of different implanted devices.

## Results

### Validation of the FE intact cervical spine model

**Fig. 4:**
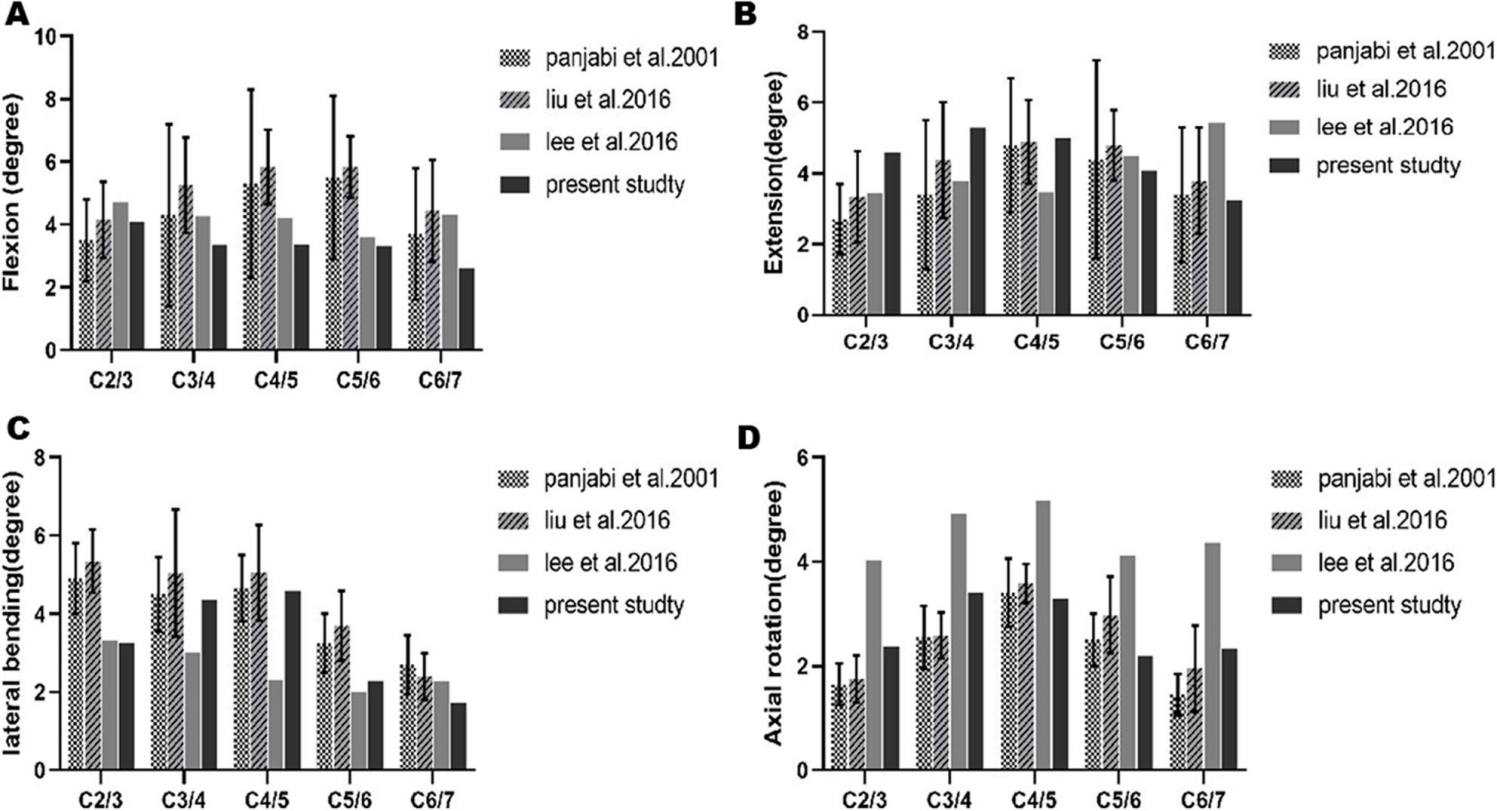
Intersegmental ROMs of the intact model are compared with those of previously published studies, (**A**) Flexion, (**B**) Extension, (**C**) Lateral bending, (**D**) Axial rotation

The segmental ROM of the intact FE model was compared with findings from previous studies, demonstrating robust consistency with both in vitro experiments and earlier finite element results [24, 31, 32] (Fig. 4A, B, C, D). Given the comprehensive comparisons with in vitro cadaver studies and FE investigations, the current FE model can be confidently considered validated and is deemed suitable for use in the present study.

### ROMs of the surgical segment and adjacent segments

The ROM for C4-6 in flexion, extension, bending, and rotation in the intact model were 5.06°, 16.34°, 10.35°, and 8.65°, respectively. Postoperatively, in the TTMC group, the ROMs for C4-6 in flexion, extension, bending, and rotation were 0.05°, 0.55°, 0.32°, and 0.33°, respectively. In the NATMC group, the corresponding ROMs for flexion, extension, bending, and rotation were 0.10°, 0.59°, 0.32°, and 0.25°, respectively. Compared with the intact model, the ROM of C4-6 in both the TTMC and NATMC surgery groups decreased by 96.91% and 96.89%. There was no significant difference between the two groups; both demonstrated excellent stability in the surgical segment.

**Fig. 5:**
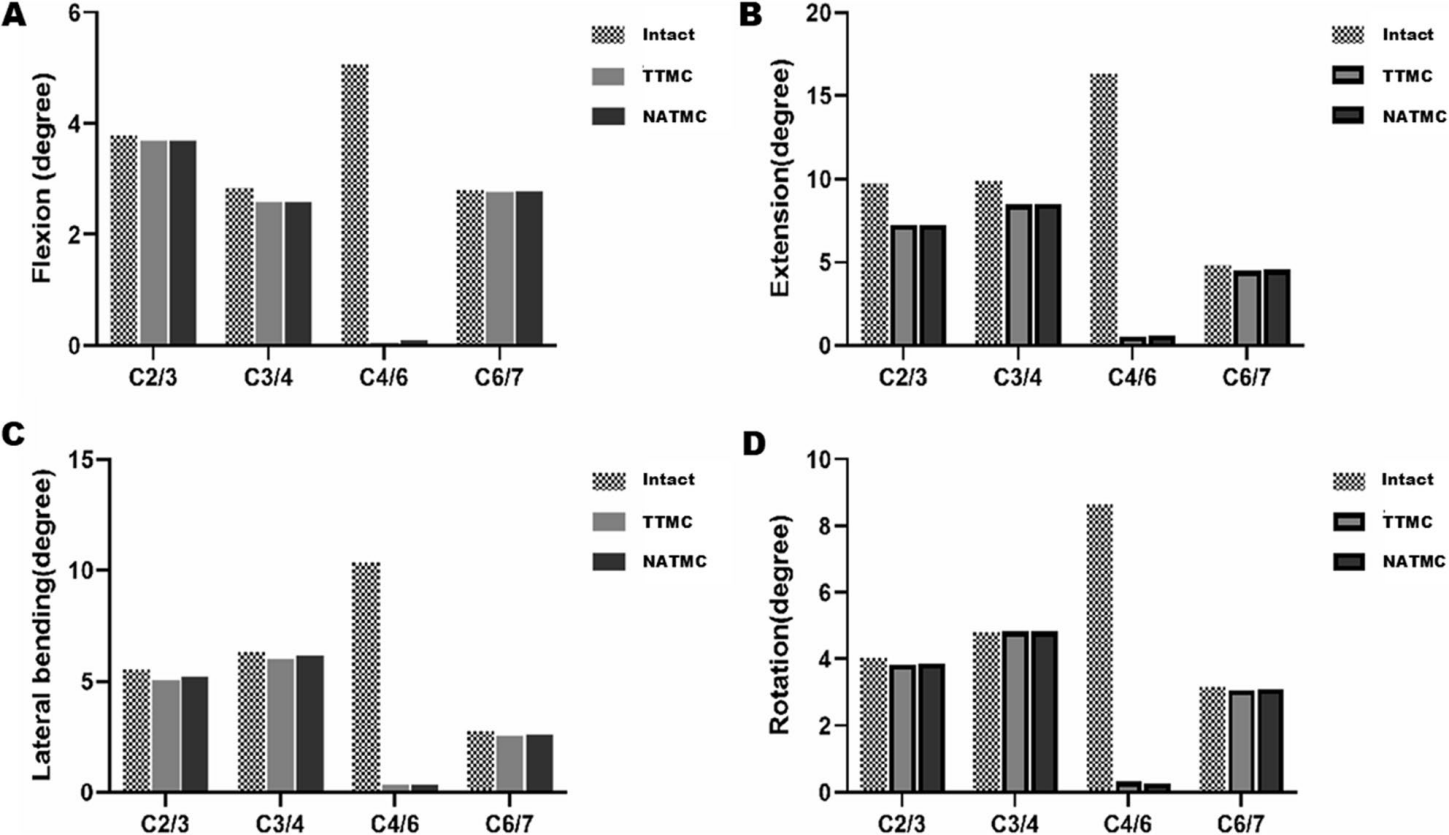
The ROMs of surgical segments (C4-C6) compared with the intact model, (**A**) Flexion, (**B**) Extension, (**C**) Lateral bending, (**D**) Rotation

In the adjacent segment at the C3/4 level, the ROMs for flexion, extension, bending, and rotation were 2.58°, 8.47°, 6.17°, and 4.83°, respectively, in the TTMC group. In the NATMC group, the corresponding ROMs were 2.58°, 8.49°, 6.18°, and 4.83°, respectively. At the C6/7 level, the ROMs for flexion, extension, bending, and rotation were 2.77°, 4.49°, 2.54°, and 3.08°, respectively, in the TTMC group. In the NATMC group, the corresponding ROMs were 2.78°, 4.56°, 2.59°, and 3.10°. When compared with the intact model data, there was no significant difference between the postoperative and intact models in the ROMs of the adjacent segment (Fig. 5A, B, C, D).

### Stress distribution on cortical endplate

**Fig. 6:**
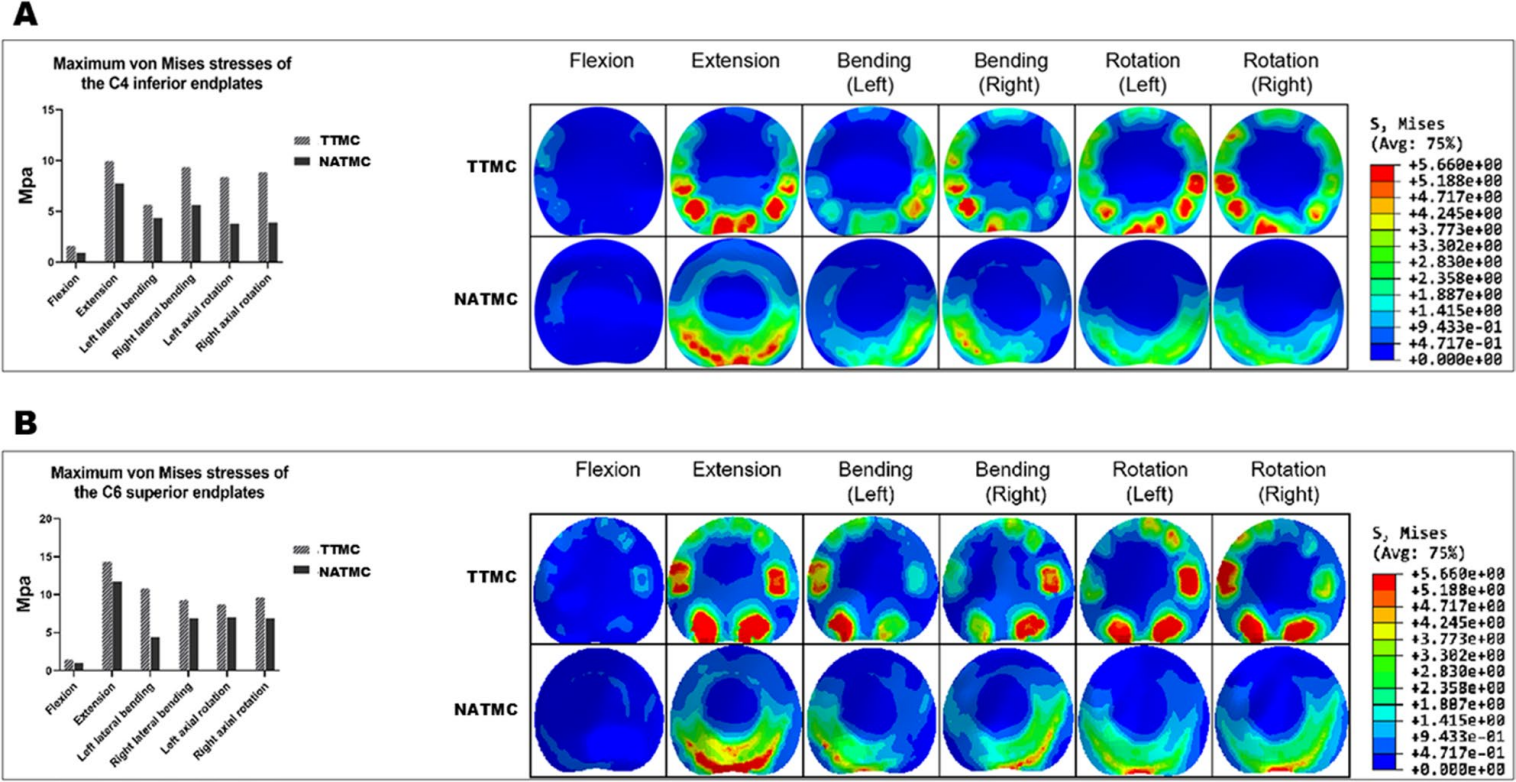
The maximum von Mises stresses of the endplate between the TTMC and NATMC models, (**A**) The C4 inferior endplate, (**B**) The C6 superior endplate

The stress distribution on the TMC-endplate interface depends on the type of fixation devices. As illustrated in Fig. 6A, in the TTMC group, the stress peaks on the C4 inferior TMC-endplate interface during flexion, extension, bending, and rotation are 1.61, 9.69, 7.52, and 8.63 MPa, respectively. In the NATMC group, the corresponding stress peaks are 0.93, 7.75, 5.00, and 3.85 MPa, respectively, in the same motion directions.

On the C6 interface of the TMC-endplate, under flexion, extension, bending, and rotation motion conditions, the stress peaks are 1.46, 14.29, 10.06, and 9.22 MPa in the TTMC model and 1.08, 11.69, 5.62, and 6.96 MPa in the NATMC model. Obviously, the TMC-endplate interface stress in the ACCF model using TTMC is significantly higher in all motion planes compared to the ACCF model using NATMC (Fig. 6B).

### Stress distribution on screw-bone interface

**Fig. 7:**
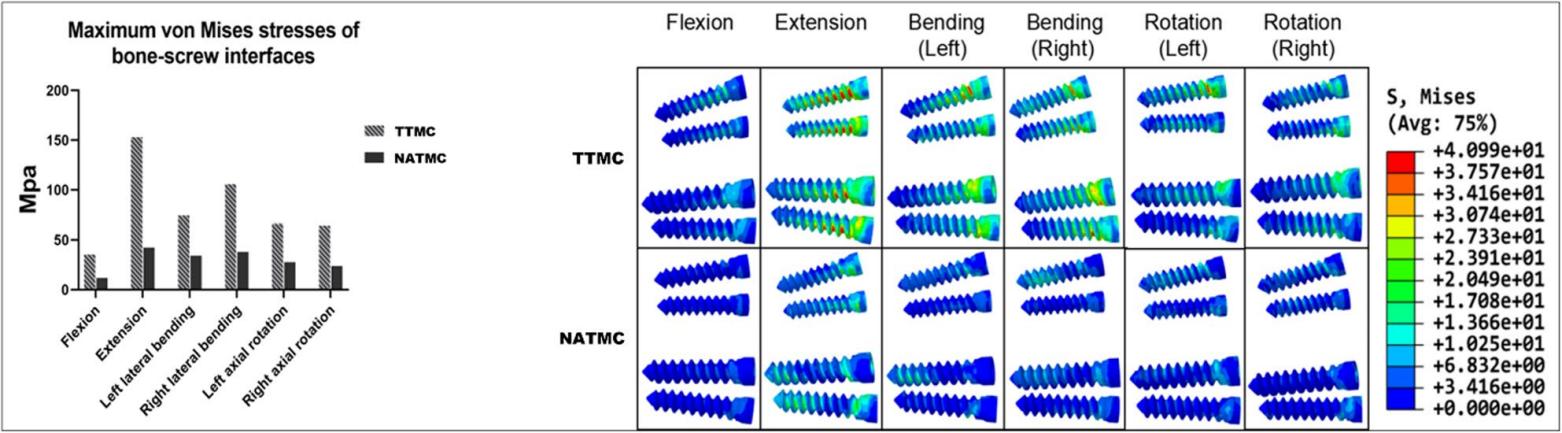
The maximum von Mises stresses on bone-screw interface between the TTMC and NATMC models

As presented in Fig. 7, the maximum von Mises stress at the screw-bone interface of C4 in the flexion, extension, lateral bending, and rotation motion directions are 35.49, 153.00, 90.19, and 86.1 MPa, respectively, and 11.91, 42.49, 36.12, and 25.85 MPa in the NATMC model, respectively. The stress cloud map illustrating the interfacial stresses between the screw and bone in both models.

### Stress distribution on adjacent intervertebral disc

**Fig. 8:**
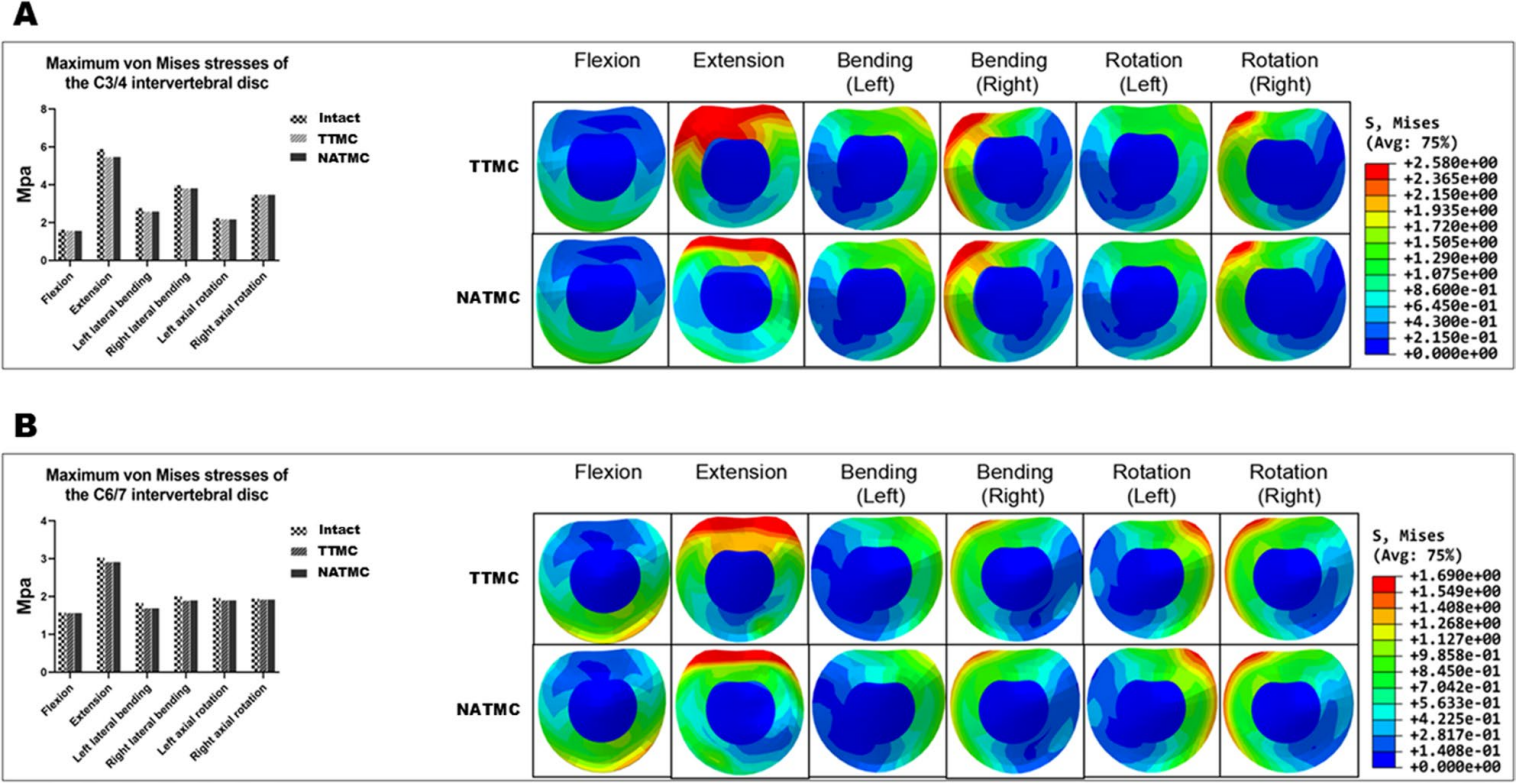
The maximum von Mises stresses in the intervertebral disc among the models, (**A**) C3/4 segment, (**B**) C6/7 segment

In Fig. 8A, the maximum stresses experienced by the C3/4 intervertebral disc in flexion, extension, lateral bending, and rotation directions were 1.56, 5.46, 3.19, and 2.82 MPa in the TTMC model and 1.56, 5.47, 3.20, and 2.82 MPa in the NATMC model, respectively. In the normal model, the maximum stresses of C3/4 were 1.62, 5.89, 3.38, and 2.85 MPa, respectively. It is evident that the utilization of both internal fixation devices did not result in an elevation of stress on the C3/4 intervertebral disc.

At the C6/7 intervertebral disc, the maximum stresses in flexion, extension, lateral flexion, and rotation directions were 1.56, 2.91, 1.79, and 1.91 MPa in the TTMC group, and 1.56, 2.91, 1.80, and 1.91 MPa in the NATMC group, respectively. Similar to C3/4, there was no significant increase in stress compared to the intact model. The stress distribution on the C6/7 disc is illustrated in Fig. 8B.

### Stress distribution on post bone

**Fig. 9:**
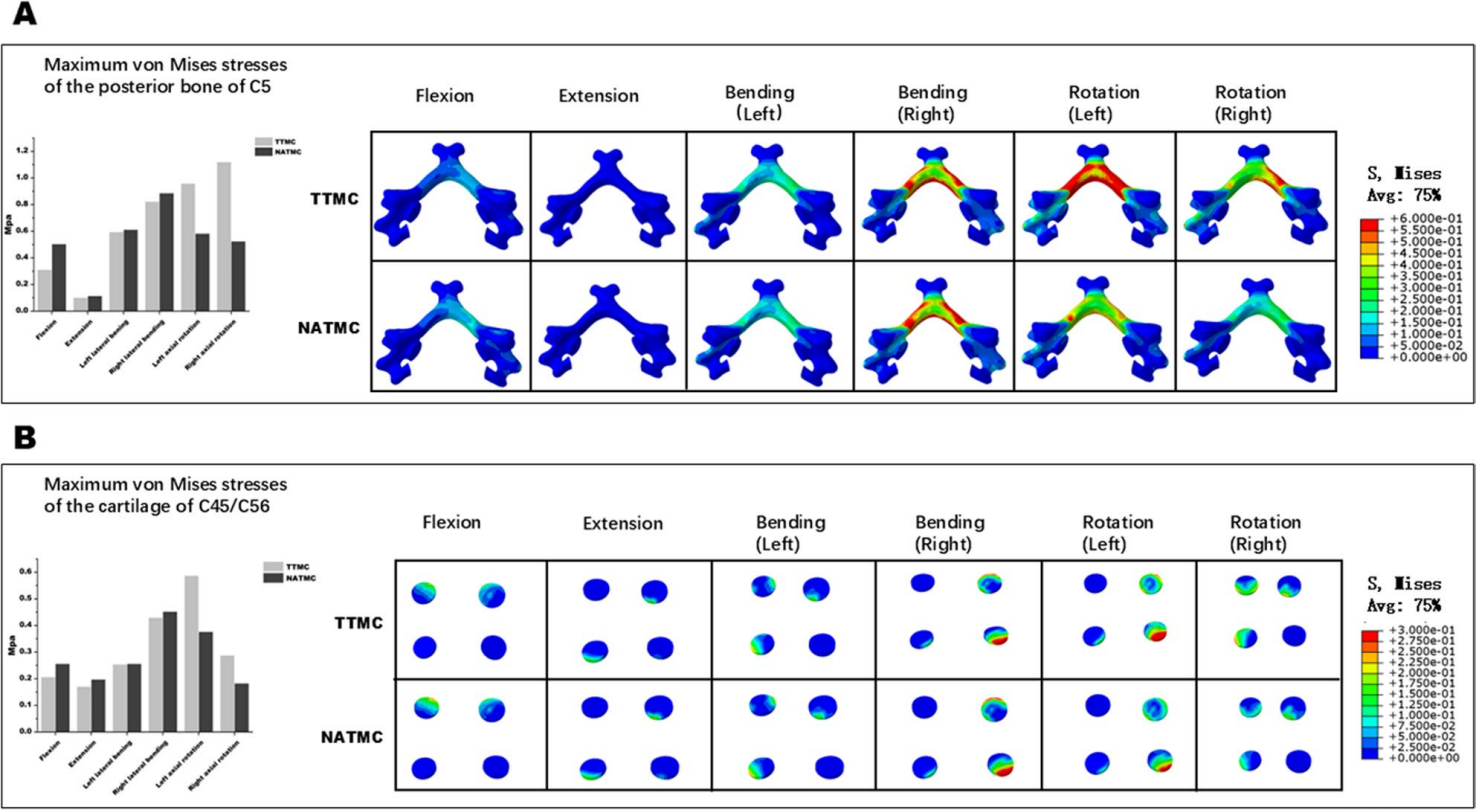
The maximum von Mises stresses in the posterior bone of C5 and cartilages of C4/5 and C5/6 among the models, (**A**) The posterior bone of C5, (**B**) Cartilages of C4/5 and C5/6

The stress distribution on post bone of C5 after two ACCF simulations was evaluated. As presented in Fig. 9A, the maximum von Mises stress at the post bone of C5 in the flexion, extension, left lateral bending, right lateral bending, left rotation and right rotation motions were 0.31, 0.10, 0.59, 0.82, 0.96, and 1.12 MPa in TTMC model, respectively, and 0.50, 0.11, 0.61, 0.89, 0.58, and 0.52 MPa in the NATMC model, respectively. The stress distribution was higher in the right lateral bending, and rotation motions.

### Stress distribution on cartilage

The stress distribution on cartilage of C4/C5 and C5/C6 after two ACCF simulations was targeted. As showed in Fig. 9B, the maximum von Mises stress at the cartilages of C4/C5 and C5/C6 in the flexion, extension, left lateral bending, right lateral bending, left rotation and right rotation motions were 0.21, 0.17, 0.25, 0.43, 0.59, and 0.29 MPa in TTMC model, respectively, and 0.26, 0.11, 0.26, 0.45, 0.38, and 0.18 MPa in the NATMC model, respectively. The stress distribution was higher in the right lateral bending, and left rotation motions.

## Discussion

The postoperative stability of the vertebral structure is crucial for the success of ACCF surgery. Previous in vitro biomechanical and FE studies have demonstrated a significant reduction in the ROM for flexion, extension, lateral flexion, and rotation of the surgical segment after ACCF [25, 33, 34]. In this study, we conducted a comprehensive comparison of the stability of models utilizing TTMC and NATMC in ACCF surgery.

Based on the results of the ROM for the surgical segment, both TTMC and NATMC models exhibited a substantial reduction (96.91%, 96.89%, respectively) in the ROM of the surgical segment (C4-6) compared to the normal model. Compared to the published reports, the ROMs of intact model in this study were similar to an intact cervical finite element simulation [35], the ROMs of ACCF were lower than the ACDF models [36], and within the failure point [37]. Thus, both TTMC and NATMC demonstrate the ability to provide effective immediate postoperative stability for ACCF, meeting the fundamental requirements of anterior cervical internal fixation.

In comparison to TTMC, NATMC boasts certain advantages in structural design. Firstly, it features a larger contact area at the titanium mesh-endplate interface, thereby enhancing local stability. Additionally, NATMC’s integrated design for the titanium mesh and vertebral fixation device efficiently disperses stress, further improving structural stability. This study, adhering to the boundary relationship between the endplate and titanium mesh interface from prior research, simulates the long-term bone fusion state after ACCF [21]. The findings suggest that NATMC not only ensures immediate postoperative stability but also provides long-term stability for ACCF surgery.

Titanium mesh subsidence is a prevalent complication following ACCF. Severe subsidence of the titanium mesh can lead to kyphosis, deterioration of neurological function, failure of internal fixation, and may even necessitate revision surgery [38, 39]. While various factors contribute to titanium mesh sinking, the primary cause is stress concentration at the interface between the titanium mesh and the endplate, resulting in the penetration of the titanium mesh through the endplate and subsequent settlement [40, 41]. The major factor leading to stress concentration is the point-to-point contact between the TTMC and the bony endplate [42]. The limited contact area between TTMC and the endplate results in sustained high stress, leading to titanium mesh penetration through the endplate and subsequent settlement. This perspective is corroborated by fusion cage studies aimed at reducing settlement rates. Similar to our novel design, Zhang et al. [20] achieved a significant reduction in peak stress at the titanium mesh-endplate interface by increasing the contact area between the titanium mesh end surface and the endplate, thereby reducing settlement rates. Wang et al. [43] improved the anatomical matching relationship between the titanium mesh and the endplate, increased stress-bearing area on the titanium mesh end surface, effectively ameliorating titanium mesh settlement. Hence, enhancing the contact relationship between the titanium mesh and the endplate is crucial for mitigating titanium mesh settlement.

In prior in vivo and in vitro investigations conducted by scholars regarding the anatomical titanium mesh, comparable findings have been reported. A cadaveric study carried out by Lu et al. demonstrated that, in contrast to the “point contact” of the traditional titanium mesh cage, the surface - contact design was capable of enhancing the uniformity of endplate stress by 40% and reducing the interfacial stress by 30% [1]. Additionally, an in - vitro experiment by Ouyang et al. further validated that, within the degenerated endplate model, the anatomical titanium mesh cage (similar to NATMC), through its curved - surface fitting design, could decrease the screw - bone interface stress by 25% [28]. This result is highly congruent with the FE outcomes of the present study, where the screw stress of NATMC was reduced by 60%.

In the realm of clinical research, a prospective cohort study led by Chen et al. revealed that the subsidence rate of the traditional titanium mesh cage stood at 15.7%, whereas for the anatomical titanium mesh cage (similar to NATMC), owing to its larger contact area, the subsidence rate dropped to 7.2% (*P* < 0.05) [15]. Furthermore, a multi - center clinical study conducted by Yin et al. further corroborated that, by adjusting the size of the auricular plate during the surgical procedure, the personalized titanium mesh cage was able to lower the postoperative screw loosening rate from 12.7% for TTMC to 4.2%, directly substantiating the stress - dispersion superiority of NATMC [12].

In this study, a Prevention of Titanium Mesh Collapse was developed, incorporating an anti-subsidence ring at the end of the titanium mesh, forming a hollow ring structure. The surface of the anti-subsidence ring establishes a face-to-face anatomical matching relationship with the endplate. In comparison to TTMC, the contact area between the end of NATMC and the endplate is significantly increased, effectively dispersing local stress and preventing excessive stress concentration. Compared with previous studies on anatomical titanium cage, this study also has obvious advantages. Although the study of Zhang et al. adopted an anti-settlement design, the two ends of the titanium mesh adopted a closed design, which hindered the possibility of bone fusion between vertebrae and may lead to long-term complications of non-fusion [20]. The titanium mesh design of Wang et al. took into account the needs of interbody fusion and adopted a hollow design at both ends of the titanium cage. However, the fixation method had drawbacks [43]. The screw fixation needed to pass diagonally through the final plate, which damaged the integrity of the end plate, and there was the possibility of internal fixation loosening and increased settlement risk. Therefore, the new titanium cage needs to be designed with anti-settlement and stability functions.

Analysis of peak stress at the titanium mesh-endplate interface revealed significantly higher stress levels in the TTMC group compared to the NATMC group under different cervical spine motion states. In the TTMC finite element model, an assumption was made that the end of the titanium mesh and the endplate surface perfectly fit, but achieving such perfection in actual surgery is nearly impossible, potentially exacerbating stress concentration. Analysis of the endplate stress indicated that the larger contact area and perfectly matched curved surface structure of NATMC result in even endplate stress distribution, reducing stress concentration and, consequently, exhibiting a lower settlement rate.

Internal fixation failure is a significant concern in ACCF, encompassing issues like screw loosening, plate fracture, and TMC dislodgement [15, 39, 44]. These complications can lead to spinal cord and nerve root compression, damage to adjacent organs, and even paralysis. While various risk factors contribute to internal fixation failure, such as osteoporosis, endplate damage during surgery, and inappropriate functional exercise, the design flaws of TTMC remain a crucial risk factor [17, 45]. Prior research indicates that excessive stress at the endplate interface, bone-screw interface is the primary cause of instrument-related complications in ACCF [20, 25].

In the present study, the NATMC model exhibited markedly lower stress levels than the TTMC model at the C4 and C6 endplate-titanium mesh interfaces. This difference was attributed to the innovative design of anti-subsidence rings, which augmented local contact area and structural conformity, resulting in a more uniform stress distribution. Additionally, at the screw-bone and screw-plate interfaces, the stress peaks in the NATMC model were also lower than those observed in the TTMC model. This indicates that the newly designed NATMC effectively disperses the load, mitigates excessive stress at the screw interface, and diminishes the risk of internal fixation failure. Although in our research, we did not compare different types of cages with anti-subsidence rings (for example: XP-CORPECTOMY CAGE, OSIMPLANT; ADD plus™, Solidity) in biomechanical properties, however, from the published similar studies [20, 46], the newly designed cages with anti-subsidence ring could alleviate the stress of endplates, intervertebral discs, and screw bone interfaces.

ACCF is recognized as a substantial risk factor for the degeneration of adjacent intervertebral discs, primarily attributed to compensatory increases in motion and heightened disc stress in segments proximal to the surgical site. Consequently, this results in compromised nutrient diffusion within the intervertebral disc, ultimately expediting the process of disc degeneration [47–50].

In this study, both the TTMC and NATMC models did not show a significant increase in motion in the adjacent surgical segments (C3/4, C6/7) compared to the normal model. Additionally, the motion in these adjacent segments was similar between the two models. Moreover, the disc stress in the adjacent segments of both the TTMC and NATMC models was also similar, with no notable increase compared to the normal model. Consequently, in comparison to traditional TTMC, NATMC does not significantly elevate motion or disc stress in adjacent segments, thereby avoiding acceleration of degeneration in the segments neighboring ACCF surgery.

This study has several limitations: (1) The finite element model was constructed using cervical CT data from healthy female subjects, neglecting gender differences and degenerative changes such as facet joint hypertrophy, osteoporosis, reduced intervertebral space height, and alterations in cervical curvature. Cervical spine models varying in age, gender, bone mass, and curvature exhibit differences in geometric morphology, mechanical responses, material property parameters, and cervical alignment—factors that collectively influence load transfer dynamics in FEA and thereby shape analytical outcomes [1, 2, 15].With respect to gender disparities, male models typically exhibit greater vertebral body height, cross-sectional area, and endplate curvature, which can alter load transfer patterns and subsequently impact FEA results [3]. Age-related variations primarily manifest in bone mechanical properties: distinct bone densities necessitate tailored elastic modulus assignments, directly influencing stress distribution at the implant-bone interface [20]. As prior research has demonstrated, anatomically designed titanium cages reduce stress at the implant-endplate interface in osteoporotic models, when contextualized with our findings, the NATMC suggests particular clinical utility for osteoporotic patients, a population prone to subsidence and fixation failure. Cervical curvature influences FEA primarily through its effect on load transfer mechanics: for example, cervical kyphosis elevates anterior stress on the titanium cage, potentially impacting its mechanical performance [4]. (2) In simulating the surgical process, achieving complete alignment between TTMC and endplate surfaces, as done in the actual surgery, proves challenging. (3) The finite element model serves as a simplification of the real cervical spine surgical model and cannot fully replicate the biomechanical environment during actual surgery, including micro-movements at the internal fixation-bone interface, muscle tension, and other factors. (4) The design and function of our NATMC structure were similar to those with anti-subsidence structures among the existing commercial products, however, the differences in biomechanical properties between different anti-subsidence ring structures were not compared, in future research, we will further compare different anti-subsidence ring products with our design using fatigue testing for the failure risk and the long-term mechanical failure analysis by combining 3D printing and specimen experiments.

## Conclusions

The findings from the finite element analysis indicate that, firstly, NATMC provides robust postoperative stability for ACCF. Secondly, NATMC effectively mitigates stress by enhancing the contact area and conformity at the cage-endplate interface, thereby preventing stress concentration and reducing the subsidence rate of the titanium mesh. Thirdly, NATMC does not significantly elevate stress in the adjacent intervertebral discs, thus averting the hastening of adjacent disc degeneration. Lastly, NATMC displays lower stress at the screw-plate interface and bone-screw interface in comparison to TTMC, contributing to a decreased likelihood of postoperative complications associated with internal fixation and promoting long-term effectiveness.

## Data Availability

The data and materials are available from the corresponding author.
